# Ciprofloxacin-Induced Oxidative Stress Promotes a Senescence-like Phenotype in Human Fibroblasts In Vitro

**DOI:** 10.3390/ijms27198696

**Published:** 2026-09-29

**Authors:** Natalia D. Kondratenko, Maria A. Chelombitko, Liudmila A. Zinovkina, Anna A. Dashkevich, Anastasia S. Prikhodko, Renata Yu. Kuptsova, Elizaveta P. Ziubina, Konstantin G. Lyamzaev, Roman A. Zinovkin

**Affiliations:** 1 Faculty of Bioengineering and Bioinformatics, Lomonosov Moscow State University, 119991 Moscow, Russia; nataliadkon@gmail.com (N.D.K.); luzinovkina@gmail.com (L.A.Z.); anna.dash.221@gmail.com (A.A.D.); rbetenkova@gmail.com (R.Y.K.); zyubina05@inbox.ru (E.P.Z.); 2 A.N. Belozersky Institute of Physico-Chemical Biology, Lomonosov Moscow State University, 119991 Moscow, Russia; atma69@yandex.ru (M.A.C.); chudoprirody@gmail.com (A.S.P.); lyamzaev@gmail.com (K.G.L.); 3 Russian Clinical Research Center for Gerontology, Pirogov Russian National Research Medical University, Ministry of Healthcare of the Russian Federation, 117513 Moscow, Russia

**Keywords:** antibiotics, fluoroquinolones, cellular senescence, fibroblasts, oxidative stress

## Abstract

Fluoroquinolones are effective synthetic broad-spectrum antibiotics, but their use can be associated with persistent adverse effects, sometimes described as fluoroquinolone-induced disability. The molecular mechanisms underlying these effects remain incompletely understood, although accumulating evidence implicates mitochondrial dysfunction, oxidative stress, and DNA damage. We hypothesized that the convergence of these stress responses may promote cellular senescence and thereby contribute to persistent fluoroquinolone-associated adverse effects. We examined three clinically used fluoroquinolones—ciprofloxacin, moxifloxacin, and levofloxacin—in primary human fibroblasts in vitro. Exposure for 7 days revealed that 200 µM ciprofloxacin, but not 200 µM moxifloxacin or 500 µM levofloxacin, induced a senescence-like phenotype characterized by increased senescence-associated β-galactosidase (SA-β-Gal) activity and an elevated senescence index (SI), which integrates increased cell size and autofluorescence measured by imaging flow cytometry. At 7 days, ciprofloxacin did not induce a broad canonical senescence-associated secretory phenotype (SASP) at either the transcriptional or secretory level, consistent with a non-canonical senescence-like state; only a modest increase in IL-1β secretion emerged after 14 days. The ciprofloxacin-induced phenotype was not accompanied by a robust increase in the canonical p53, p16, or p21 protein markers, but was associated with reduced retinoblastoma protein (RB) phosphorylation at Ser795. However, ciprofloxacin removal from the culture medium after 1 or 2 weeks of incubation resulted in the recovery of the resazurin signal to approximately control values by the end of the 3-week experimental period. Ciprofloxacin also increased oxidative stress, as assessed using dihydroethidium and dichlorodihydrofluorescein diacetate. Both the antioxidant Trolox and the mitochondria-targeted antioxidant SkQ3 attenuated the ciprofloxacin-induced increase in SA-β-Gal activity and SI. These findings identify oxidative stress as a putative mediator of ciprofloxacin-induced senescence-like remodeling in human fibroblasts and support further investigation of drug-induced cellular senescence as a potential contributor to persistent fluoroquinolone-associated adverse effects.

## 1. Introduction

Fluoroquinolones are synthetic broad-spectrum antibacterial agents widely used to treat bacterial infections. Their antibacterial activity is mediated primarily by inhibition of bacterial DNA gyrase and topoisomerase IV, enzymes required for DNA replication and transcription [1,2]. Despite their clinical efficacy, systemic fluoroquinolone use is constrained by safety concerns. Reported adverse effects include tendinopathy and tendon rupture, musculoskeletal pain, muscle weakness, peripheral neuropathy, gastrointestinal symptoms, and neuropsychiatric manifestations [3,4,5]. Some patients develop persistent or delayed symptoms after fluoroquinolone exposure, a condition sometimes referred to as fluoroquinolone-induced disability [6].

Experimental evidence indicates that mitochondrial dysfunction and oxidative stress are major components of fluoroquinolone toxicity at the cellular level. Mitochondria may be particularly susceptible to fluoroquinolones because of their bacterial evolutionary origin and the presence of mitochondrial topoisomerases. Ciprofloxacin (CIP), moxifloxacin (MOX), and levofloxacin (LEV) have been reported to induce cell death, increase reactive oxygen species (ROS), and elevate oxidative stress markers such as 4-hydroxynonenal [7,8]. Moxifloxacin and ciprofloxacin can reduce mitochondrial membrane potential, whereas levofloxacin has been shown to impair respiratory-chain complexes I and III and decrease cellular ATP levels [7,8,9].

The contribution of oxidative stress to fluoroquinolone toxicity is further supported by studies demonstrating protective effects of antioxidants. Conventional antioxidants, including vitamin C, vitamin E, and N-acetylcysteine, as well as the mitochondria-targeted antioxidant MitoQ, attenuate fluoroquinolone-induced ROS accumulation, mitochondrial dysfunction, lipid peroxidation, and cell death in experimental models [7,8,10,11]. The downstream cellular programs activated by fluoroquinolone-induced oxidative stress, however, remain insufficiently characterized.

Persistent mitochondrial dysfunction and oxidative stress can promote cellular senescence (reviewed in [12]). Cellular senescence is a stable cell-cycle arrest accompanied by extensive changes in cell morphology, metabolism, gene expression, and secretory activity. Common features include increased senescence-associated β-galactosidase (SA-β-Gal) activity, enlarged cell morphology, increased lysosomal mass, altered autofluorescence, activation of the p53/p21WAF1/CIP1 and/or p16INK4A/RB pathways, and, in many contexts, development of a heterogeneous senescence-associated secretory phenotype (SASP) characterized by the release of inflammatory cytokines, chemokines, growth factors, and proteases [13,14,15].

The effects of fluoroquinolones on cellular senescence remain poorly understood. Antibiotics outside this class have been reported to modulate senescence: cephalosporins can promote senescence, whereas azithromycin has shown senolytic activity in selected experimental systems (reviewed in [16]). Previous studies have reported senescence-associated effects of ciprofloxacin in specific experimental systems, including glioblastoma cells [17], and, more recently, ciprofloxacin-induced placental senescence in a mouse model [18]. However, it remains unclear whether prolonged fluoroquinolone exposure could induce a senescence-like phenotype in primary human fibroblasts, whether this response represents a general property of clinically used fluoroquinolones, and whether redox-modulating interventions could attenuate the phenotype.

In the present study, we investigated the effects of three clinically used fluoroquinolones—ciprofloxacin, moxifloxacin, and levofloxacin—on cellular senescence in human fibroblasts in vitro. These three antibiotics are members of the same antibiotic class but differ in chemical structure, cellular disposition, cytotoxic potency, and reported mitochondrial and oxidative effects. We used prolonged exposure to fluoroquinolones to model cellular stress and assessed SA-β-Gal activity, cell size and autofluorescence, SASP-associated gene expression and cytokine secretion, DNA damage response, and oxidative stress. We also tested whether conventional and mitochondria-targeted antioxidants could attenuate fluoroquinolone-induced senescence-like changes. Our results demonstrate that ciprofloxacin, but not moxifloxacin or levofloxacin, induces a non-canonical senescence-like phenotype associated with oxidative stress and sensitive to antioxidant intervention.

## 2. Results

### 2.1. Ciprofloxacin Induces a Senescence-like Phenotype in Human Fibroblasts In Vitro

**Figure 1:**
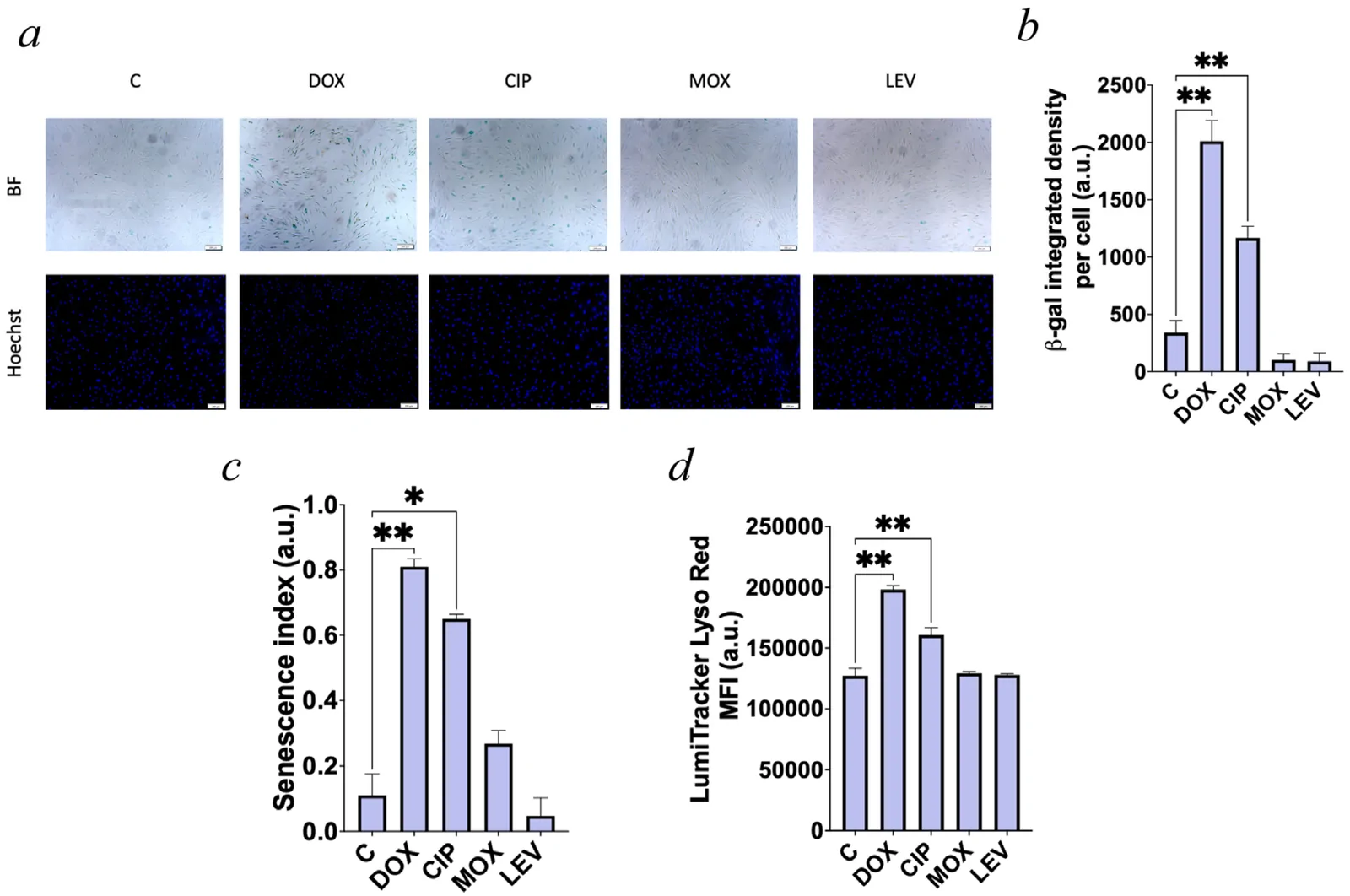
Effects of ciprofloxacin (CIP), moxifloxacin (MOX), and levofloxacin (LEV) on cellular senescence markers. (**a**,**b**) Senescence-associated β-galactosidase (SA-β-Gal) activity: (**a**) Representative images of SA-β-Gal and Hoechst staining in primary human fibroblasts treated for 7 days with 200 μM CIP, 200 μM MOX, or 500 μM LEV. Doxorubicin (DOX; 250 nM) was used as a positive control for senescence induction. Bright-field (BF) and Hoechst images are shown. Cells with no treatment indicated as control (C). Scale bar: 200 µm; (**b**) Quantification of SA-β-Gal staining as integrated density per cell, normalized to the nuclear count. Images were analyzed using ImageJ2 (Version: 2.16.0/1.54p); (**c**) senescence index (SI), calculated as SI = ((nAF − 1) + 5 × (nD − 1))/2, where nAF and nD represent autofluorescence and cell diameter, respectively, normalized to the mean values of proliferating control cells, as described in [19]; (**d**) LumiTracker Lyso Red mean fluorescence intensity (MFI) as an indicator of lysosomal mass. Data are presented as mean ± SEM from n = 3 independent biological replicates per group. For SA-β-Gal quantification, three fields per condition per replicate were averaged to obtain one value per biological replicate. Statistical comparisons were performed using one-way ANOVA followed by Dunnett’s post hoc test (each treatment vs. control); * *p* < 0.05, ** *p* < 0.01.

To investigate the effects of the selected fluoroquinolones on cellular senescence, we used 200 μM CIP, 200 μM MOX, and 500 μM LEV. These concentrations were selected on the basis of the resazurin reduction assay after 7 days of exposure in primary human fibroblasts (Supplementary Figure S1). CIP, but not MOX or LEV, significantly increased SA-β-Gal activity per cell, the senescence index (SI), which reflects cellular autofluorescence and cell size, and lysosomal content (Figure 1a–d). MOX and LEV produced a modest reduction in relative SA-β-Gal activity but did not affect SI or lysosomal content (Figure 1a–d). We have additionally tested 25, 50, 100, 200 and 400 μM CIP on human fibroblasts and found that only 200 and 400 μM increased SA-β-Gal activity (Supplementary Figure S2). 

We next examined whether CIP similarly affects SA-β-Gal activity in immortalized cell lines used in senescence research. CIP increased SA-β-Gal activity in the human endothelial EA.hy926 cell line (Supplementary Figure S3a,b) and in H9c2 rat cardiomyoblasts (Supplementary Figure S3c,d).

**Figure 2:**
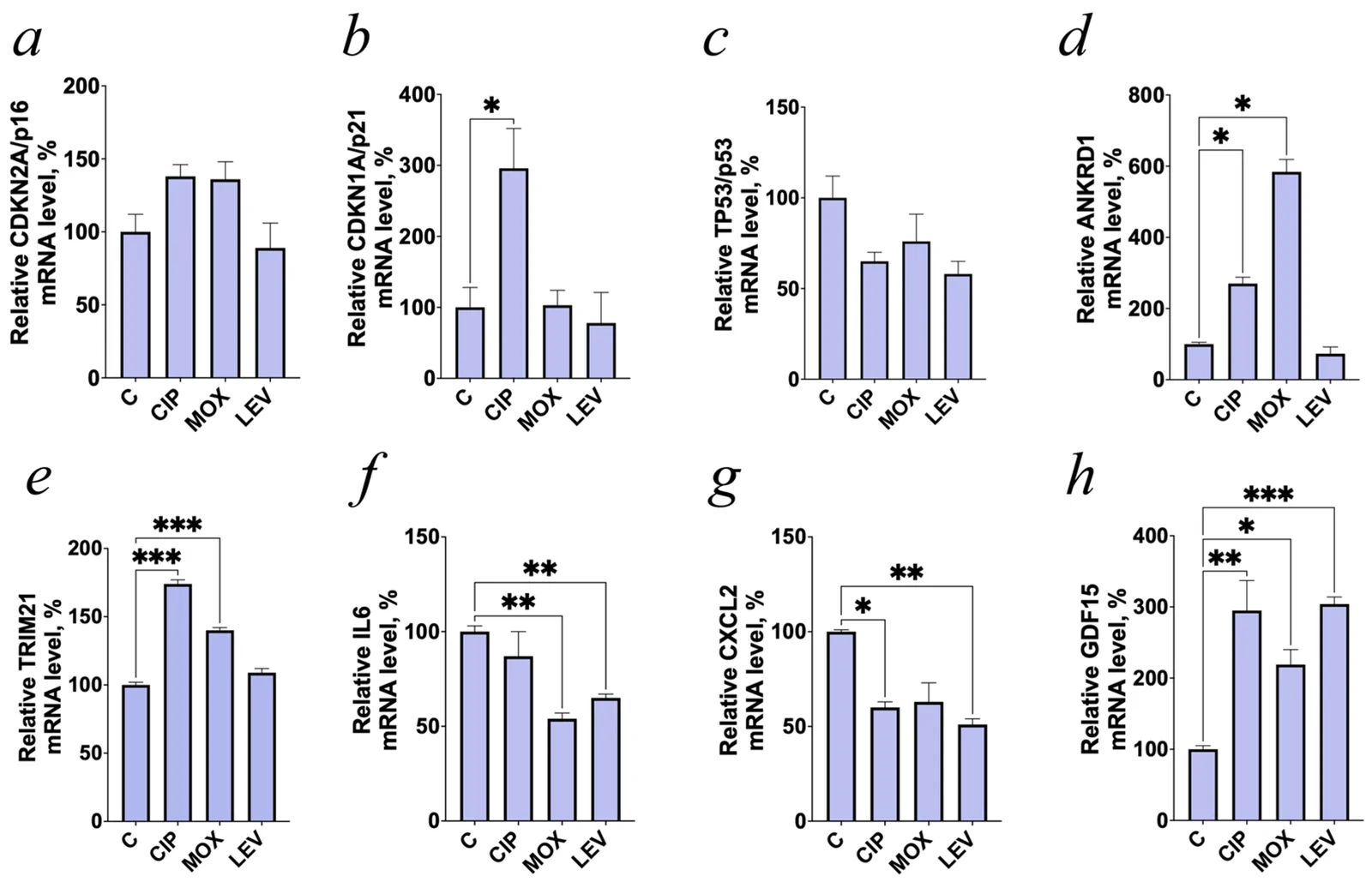
Effects of CIP, MOX, and LEV on mRNA expression of cell-cycle-regulatory, cytokine-related, and stress-response markers. Cell treatment conditions are described in the legend to Figure 1. Relative mRNA levels of (**a**) p16 (CDKN2A); (**b**) p21 (CDKN1A); (**c**) p53 (TP53); (**d**) ANKRD1; (**e**) TRIM21; (**f**) IL6; (**g**) CXCL2; and (**h**) GDF15 were quantified by RT-qPCR using the ΔCt method and normalized to the geometric mean of three reference genes (GAPDH, ACTB, and RPL32). For each target gene, the relative mRNA level in control samples was set to 100%. Data are presented as mean ± SEM from n = 3 independent biological replicates per group. Each sample was analyzed in triplicate (technical replicates), averaged to one value per biological replicate. Statistical comparisons were performed using one-way ANOVA followed by Dunnett’s post hoc test (each treatment vs. control); * *p* < 0.05, ** *p* < 0.01, *** *p* < 0.001.

We then analyzed mRNA expression of senescence-associated stress-response and cell-cycle-regulatory genes in human fibroblasts, including ANKRD1, TRIM21, CDKN2A/p16, CDKN1A/p21, and TP53/p53, together with cytokine- and stress-related genes GDF15, IL1B, IL6, TNF, CXCL2, and CXCL8/IL8 (Figure 2). For IL1B and TNF, RT-qPCR Ct values exceeded 33 cycles, precluding reliable quantification of transcript levels.

Among the cell-cycle-regulatory markers, CIP significantly increased CDKN1A/p21 expression by approximately 3-fold (*p* < 0.05), whereas MOX and LEV had no detectable effect on this gene (Figure 2b). CDKN2A/p16 expression showed a moderate upward trend after CIP and MOX treatment, but neither change reached statistical significance (Figure 2a). TP53/p53 expression was not increased by any of the tested fluoroquinolones and instead tended to decrease after treatment (Figure 2c).

The largest transcriptional changes occurred in stress-response genes. CIP increased ANKRD1 expression by approximately 2.5–3-fold, whereas MOX produced an approximately 6-fold increase; LEV had no significant effect (Figure 2d). TRIM21 expression also increased, most prominently after CIP treatment, which elevated TRIM21 mRNA by approximately 1.7-fold (*p* < 0.001). MOX induced a smaller but significant increase, whereas LEV had little or no effect (Figure 2e). GDF15 expression increased after treatment with all three fluoroquinolones, reaching approximately 2–3 times the control level (Figure 2h).

Fluoroquinolone treatment did not upregulate expression of the analyzed pro-inflammatory cytokine genes. IL6 expression was significantly reduced after MOX and LEV exposure (Figure 2f), while CXCL2 expression decreased after CIP and LEV treatment, with a similar downward trend after MOX exposure (Figure 2g). Thus, although CIP induced several stress- and senescence-associated genes, including CDKN1A/p21, ANKRD1, TRIM21, and GDF15, none of the tested fluoroquinolones elicited a broad pro-inflammatory SASP-like transcriptional response under these conditions (Figure 2).

**Figure 3:**
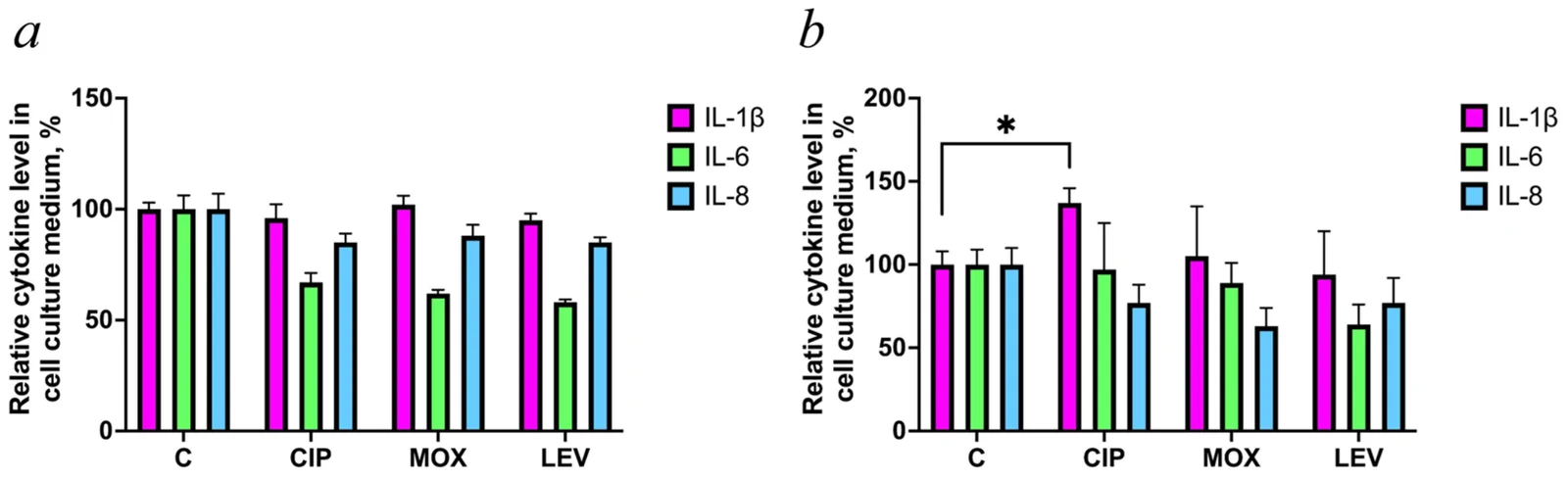
Effects of CIP, MOX, and LEV on cytokine secretion. Primary human fibroblasts were treated with 200 μM CIP, 200 μM MOX, or 500 μM LEV for (**a**) 7 days or (**b**) 14 days. Secreted IL-1β, IL-6, and IL-8 levels were measured in conditioned medium by enzyme-linked immunosorbent assay (ELISA). Data are presented as mean ± SEM from n = 3 independent biological replicates per group. Statistical comparisons were performed separately for each cytokine using one-way ANOVA followed by Dunnett’s post hoc test (each treatment vs. control); * *p* < 0.05.

We next determined whether the transcriptional changes were accompanied by increased secretion of SASP-associated cytokines. After 7 days of exposure to the selected antibiotics, secretion of IL-1β, IL-6, and CXCL8/IL-8 remained largely unchanged (Figure 3a). After 14 days of CIP treatment, however, IL-1β secretion increased modestly by approximately 1.3-fold (*p* < 0.05) (Figure 3b), indicating delayed induction of at least one SASP component.

### 2.2. Ciprofloxacin Induces DNA Damage Response and Oxidative Stress in Human Fibroblasts In Vitro

**Figure 4:**
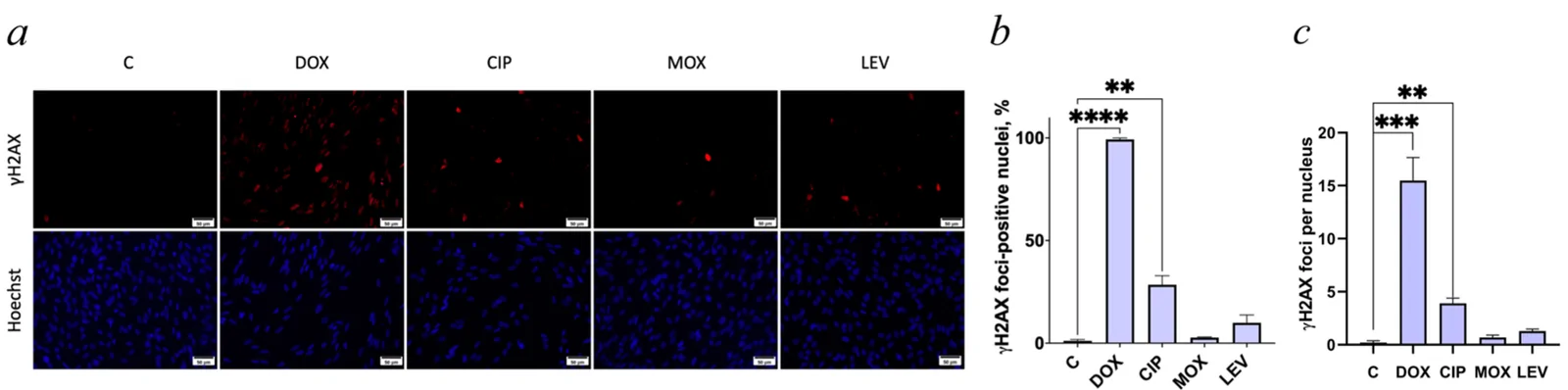
γH2AX accumulation induced by CIP, MOX, and LEV. Cell treatment conditions are described in the legend to Figure 1. (**a**) Representative images of γH2AX immunofluorescence (red) and Hoechst nuclear staining (blue) in the same fields. Scale bar: 50 µm; (**b**) quantification of γH2AX-positive cells per field. Positive cells were identified by visual scoring of nuclei with γH2AX staining clearly above background; (**c**) quantification of the mean number of γH2AX foci per nucleus across all nuclei analyzed. Data are presented as mean ± SEM from n = 3 independent biological replicates per group. For γH2AX quantification, 10 fields per condition per replicate were averaged to obtain one value per biological replicate. Statistical comparisons were performed using one-way ANOVA followed by Dunnett’s post hoc test (each treatment vs. control). ** *p* < 0.01, *** *p* < 0.001, **** *p* < 0.0001 vs. control.

Because fluoroquinolones can inhibit cellular topoisomerases in vitro and induce DNA damage [20], we assessed phosphorylation of histone H2AX at Ser139 (γH2AX), a widely used marker of the DNA damage response, particularly to DNA double-strand breaks. CIP significantly increased both the proportion of γH2AX-positive cells and the mean number of γH2AX foci per nucleus (*p* < 0.01 for both comparisons), whereas MOX and LEV produced no significant effects (Figure 4).

**Figure 5:**
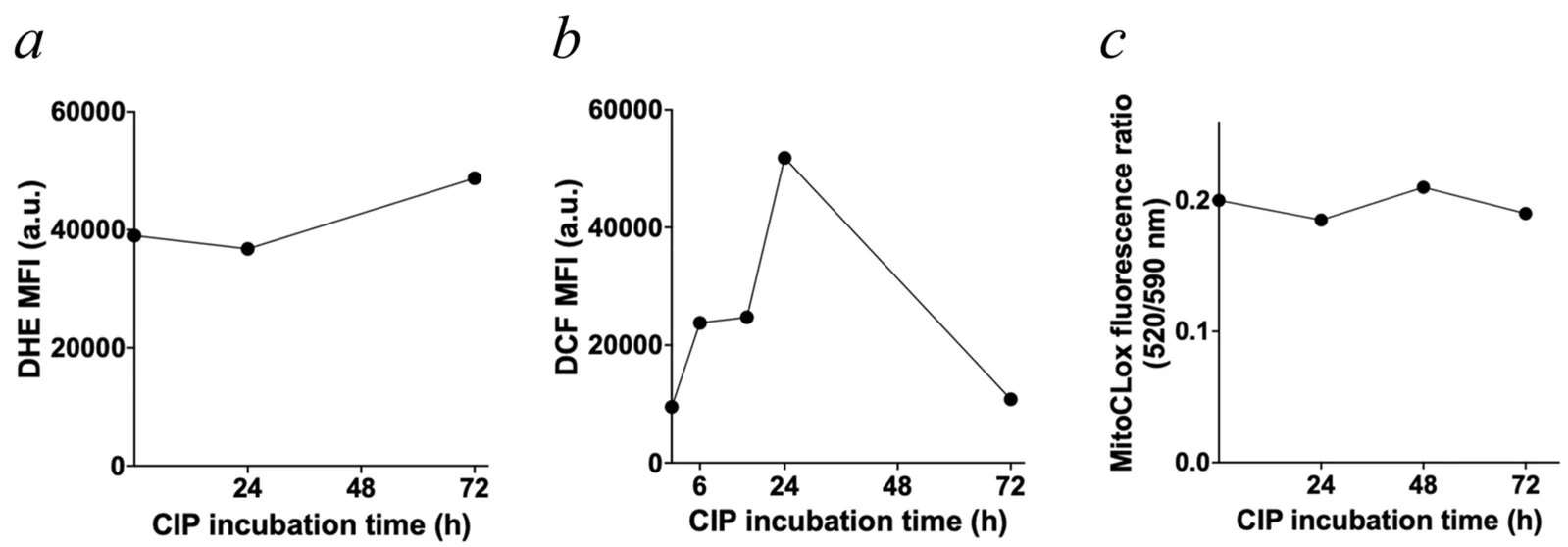
Time course of oxidative stress-related fluorescence signals in 200 μM CIP-treated human fibroblasts. Cell treatment conditions are described in the legend to Figure 1. Time-dependent changes induced by CIP were assessed using (**a**) DHE fluorescence as an indicator of superoxide-associated oxidative stress; (**b**) DCF fluorescence as a general redox-sensitive signal; and (**c**) the MitoCLox fluorescence ratio (520/590 nm) as an indicator of mitochondrial lipid peroxidation. Fluorescence signals were measured at the indicated time points after CIP exposure. Results of a single experiment are presented.

We next evaluated the temporal development of oxidative stress in CIP-treated human fibroblasts using the redox-sensitive probes dihydroethidium (DHE) and 2′,7′-dichlorodihydrofluorescein diacetate (DCFH-DA), together with the MitoCLox fluorescence ratio (520/590 nm) as a measure of mitochondrial lipid peroxidation. A preliminary time-course experiment showed that the DHE signal increased after 72 h of CIP exposure (Figure 5a), whereas the DCF signal increased earlier, after 6 h of incubation (Figure 5b). The MitoCLox fluorescence ratio remained unchanged (Figure 5c). Based on these kinetics, we selected 72 h for DHE measurements and 24 h for DCF measurements in subsequent experiments.

### 2.3. Antioxidants Attenuate CIP-Induced DNA Damage Response, Oxidative Stress, and the Senescence-like Phenotype in Human Fibroblasts In Vitro

**Figure 6:**
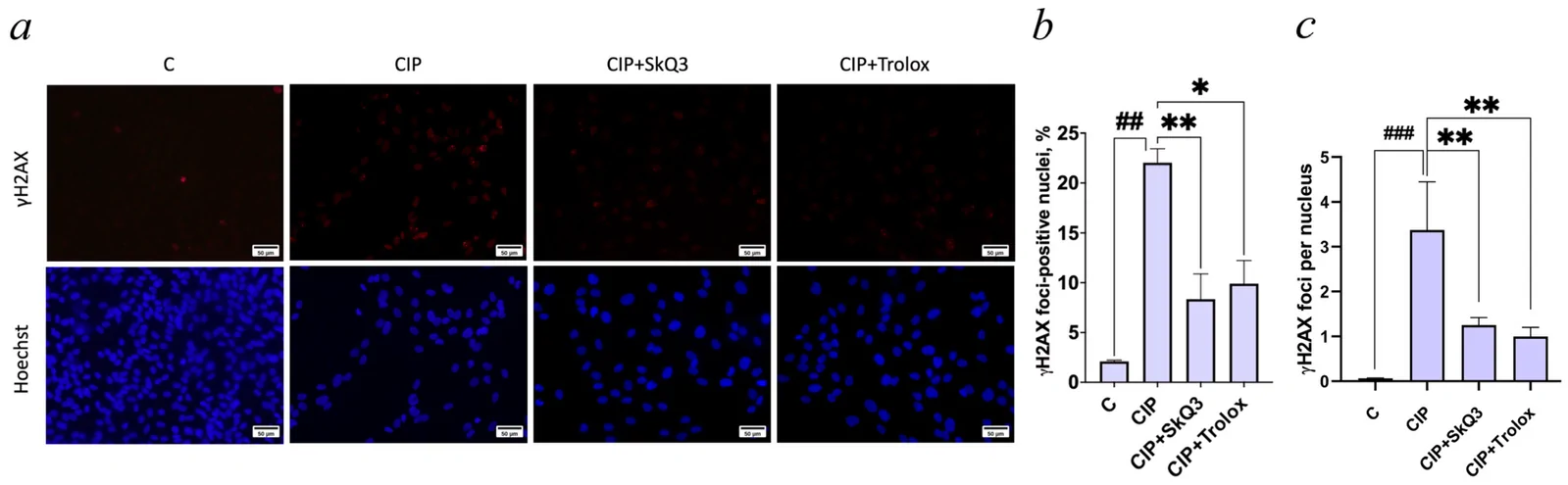
Antioxidant co-treatment attenuates CIP-induced DNA damage response assessed by γH2AX immunostaining. Human fibroblasts were treated for 7 days with 200 μM CIP alone or together with the mitochondria-targeted antioxidant SkQ3 (80 nM) or the water-soluble vitamin E analog Trolox (100 μM). (**a**) Representative images of γH2AX immunofluorescence (red) and Hoechst nuclear staining (blue) in the same fields. Scale bar: 50 µm; (**b**) quantification of the percentage of γH2AX-positive nuclei. Positive cells were identified by visual scoring of nuclei with γH2AX staining clearly above background; (**c**) quantification of the mean number of γH2AX foci per nucleus across all nuclei analyzed. Ten randomly selected fields of view were analyzed in each experimental group. Data are presented as mean ± SEM from n = 3 independent biological replicates per group. Statistical analysis was performed using one-way ANOVA followed by Dunnett’s post hoc test for comparisons with untreated control cells (^##^ *p* < 0.01, ^###^ *p* < 0.001) and Bonferroni-corrected comparisons with CIP-treated cells (* *p* < 0.05, ** *p* < 0.01).

We examined whether antioxidant co-treatment could attenuate CIP-induced DNA damage response. After 7 days, CIP increased the proportion of γH2AX-positive nuclei from approximately 2% in control cells to approximately 20–22% (Figure 6a,b). Co-treatment with 80 nM SkQ3 or 100 μM Trolox reduced γH2AX positivity to approximately 10–12%, indicating attenuation of the CIP-induced γH2AX response. Consistently, CIP markedly increased the mean number of γH2AX foci per nucleus, whereas both SkQ3 and Trolox significantly reduced this increase (Figure 6c). Thus, both the mitochondria-targeted antioxidant SkQ3 and the water-soluble antioxidant Trolox attenuated CIP-induced γH2AX accumulation in human fibroblasts.

**Figure 7:**
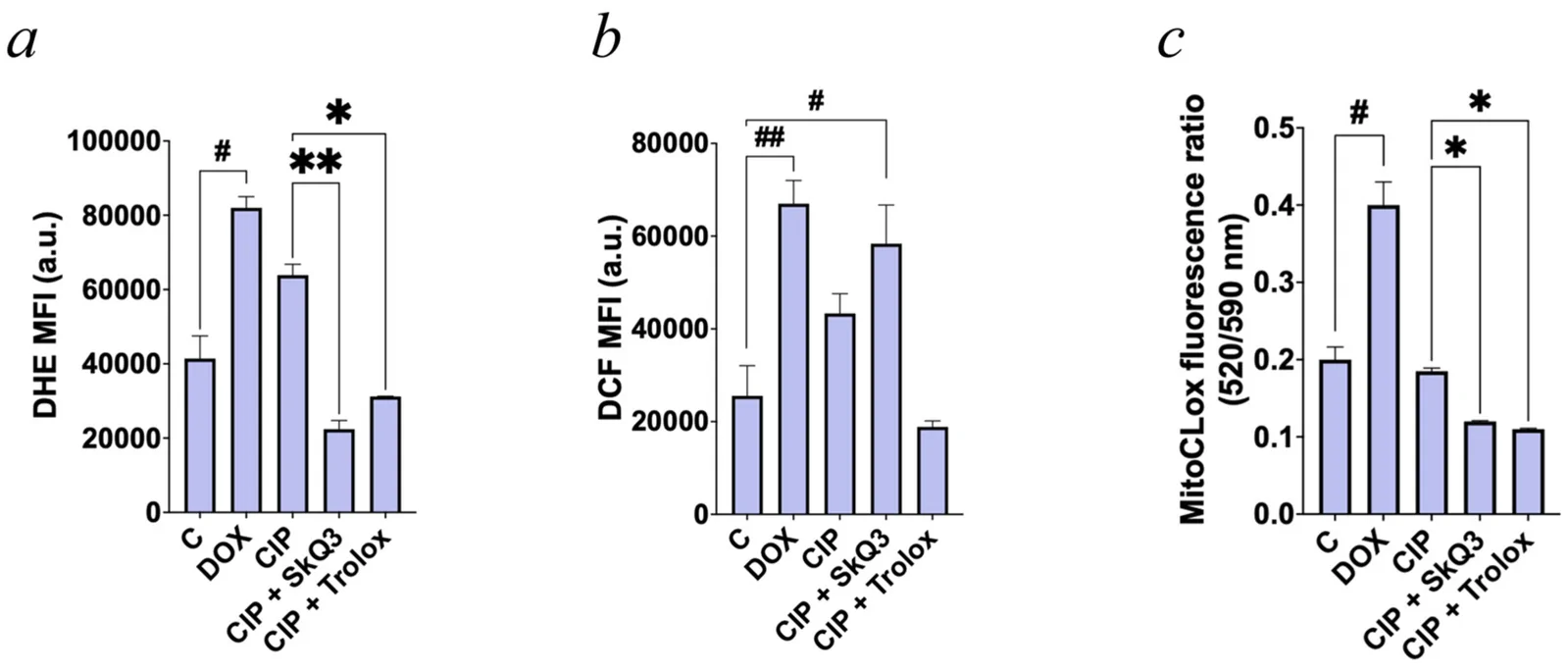
Effects of antioxidant co-treatment on oxidative stress-related fluorescence signals in ciprofloxacin-treated human fibroblasts. Human fibroblasts were exposed to 200 μM CIP alone or together with the mitochondria-targeted antioxidant SkQ3 (80 nM) or the water-soluble vitamin E analog Trolox (100 μM). Doxorubicin (DOX; 250 nM) was used as a positive control. Oxidative stress-related fluorescence was measured using (**a**) DHE after 72 h of treatment, (**b**) DCFH-DA after 24 h of treatment, and (**c**) the MitoCLox fluorescence ratio (520/590 nm) after 72 h of treatment. Data are presented as mean ± SEM from n = 3 independent biological replicates per group. Statistical analysis was performed using one-way ANOVA followed by Dunnett’s post hoc test for comparisons with untreated control cells (^#^ *p* < 0.05, ^##^ *p* < 0.01) and Bonferroni-corrected comparisons with CIP-treated cells (* *p* < 0.05, ** *p* < 0.01).

We next examined the effects of antioxidant co-treatment on CIP-induced oxidative stress (Figure 7). After 72 h, CIP increased DHE fluorescence by approximately 1.5-fold, consistent with increased superoxide-associated oxidative stress (Figure 7a). Co-treatment with either Trolox (100 μM) or the mitochondria-targeted antioxidant SkQ3 (80 nM) reduced DHE fluorescence by approximately 2-fold relative to CIP-treated cells (*p* < 0.05), resulting in values slightly below the untreated control level. DCF fluorescence also increased after 24 h of CIP exposure, but the two antioxidants had different effects on this signal. Trolox reduced the CIP-induced increase in DCF fluorescence to approximately the control level, whereas SkQ3 further increased DCF fluorescence (Figure 7b).

We also assessed mitochondrial lipid oxidation using MitoCLox. CIP did not significantly alter the MitoCLox signal after either 24 or 72 h of exposure (Figure 5c), indicating that the oxidative stress detected by DHE and DCFH-DA was not accompanied by a measurable increase in mitochondrial lipid peroxidation under these conditions. Both SkQ3 and Trolox, however, reduced the basal MitoCLox signal, consistent with lower steady-state levels of oxidized mitochondrial lipids (Figure 7c).

**Figure 8:**
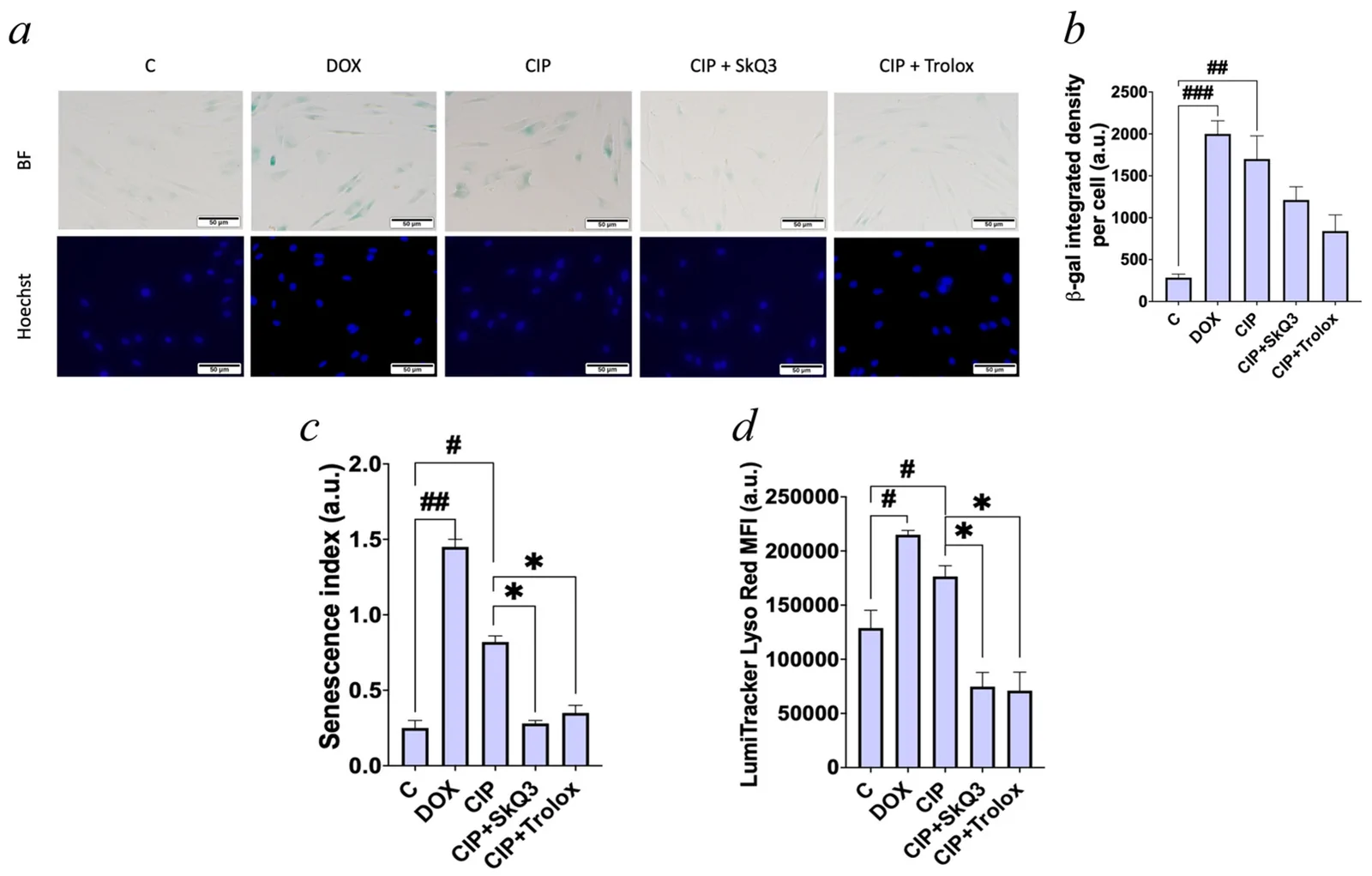
Antioxidant co-treatment attenuates CIP-induced senescence-like changes in human fibroblasts. Cell treatment conditions are described in the legend to Figure 6. Doxorubicin (DOX; 250 nM) was used as a positive control for senescence induction. (**a**) Representative images of SA-β-Gal staining under the indicated treatment conditions. The upper row shows bright-field images of SA-β-Gal-positive cells, and the lower row shows nuclear counterstaining. Scale bar: 50 μm; (**b**) quantification of SA-β-Gal integrated density per cell. Images were analyzed using ImageJ2; (**c**) senescence index calculated from imaging flow cytometry parameters; and (**d**) lysosomal content assessed by LumiTracker Lyso Red fluorescence intensity. Data are presented as mean ± SEM from n = 3 independent biological replicates. Statistical analysis was performed using one-way ANOVA followed by Dunnett’s post hoc test for comparisons with untreated control cells (^#^ *p* < 0.05, ^##^ *p* < 0.01, ^###^ *p* < 0.001) and Bonferroni-corrected comparisons with CIP-treated cells (* *p* < 0.05).

We next tested whether antioxidant co-treatment could attenuate CIP-induced senescence-like changes. Both DOX and CIP increased SA-β-Gal staining, whereas co-treatment with SkQ3 or Trolox reduced SA-β-Gal staining in CIP-treated cells (Figure 8a,b). CIP also markedly increased the senescence index, and both antioxidants reduced this parameter to values close to the untreated control level (Figure 8c). LumiTracker Lyso Red fluorescence increased significantly after CIP treatment, while co-treatment with SkQ3 or Trolox produced a downward trend (Figure 8d). Collectively, these data indicate that antioxidant co-treatment attenuates multiple features of CIP-induced senescence-like remodeling in human fibroblasts.

It should be noted that the data shown in Figure 8 were derived from an independent experimental series (different donor fibroblast preparation and imaging session) from those presented in Figure 1. Accordingly, absolute values of integrated density and senescence index differ between the two figures, reflecting inter-experiment biological variability; however, the relative pattern—CIP and DOX increasing SA-β-Gal activity and SI compared to untreated control—is consistent across both datasets.

**Figure 9:**
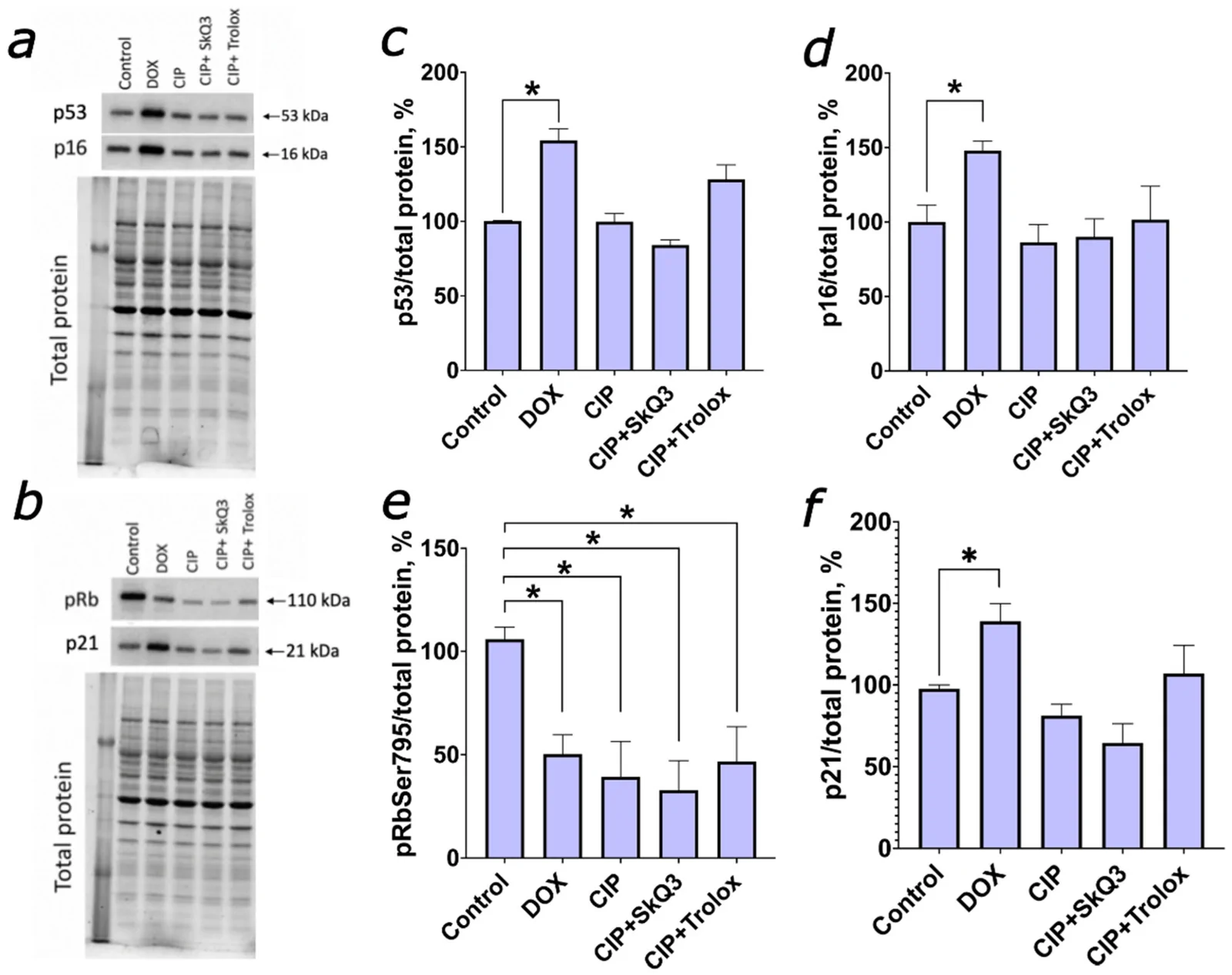
Effects of CIP and antioxidant co-treatment on senescence-associated protein markers in human fibroblasts. Cell treatment conditions are described in the legend to Figure 8. (**a**,**b**) Representative immunoblots for each target protein (upper panels) and the corresponding 2,2,2-trichloroethanol (TCE) fluorescence images of the same gels used as total protein loading controls (lower panels, “Total protein”). Densitometric quantification of (**c**) p53, (**d**) p16, (**e**) pRB Ser795, and (**f**) p21. Each protein signal was normalized to the corresponding total protein lane (TCE fluorescence) and expressed as a percentage of the untreated control. Data are presented as mean ± SEM from n = 3 independent biological replicates per group. Statistical comparisons were performed using one-way ANOVA followed by Dunnett’s post hoc test (each treatment vs. control). * *p* < 0.05 vs. control.

Western blotting was used to determine whether CIP-induced senescence-like changes were accompanied by alterations in canonical senescence-associated cell-cycle regulators (Figure 9). CIP did not increase p53, p16, or p21 protein levels relative to untreated control cells. Trolox co-treatment produced a tendency toward higher p53, p16, and p21 levels, whereas SkQ3 did not show a similar effect. DOX, used as a positive control, increased all these proteins. In contrast, RB phosphorylation at Ser795 decreased after CIP treatment and was further reduced in cells treated with CIP plus SkQ3 as well as in cells treated with DOX alone. These data indicate that CIP-induced senescence-like remodeling occurs without a robust increase in p53, p16, or p21 protein abundance but could be associated with reduced RB phosphorylation.

### 2.4. Cell Density Recovery After Ciprofloxacin Withdrawal

**Figure 10:**
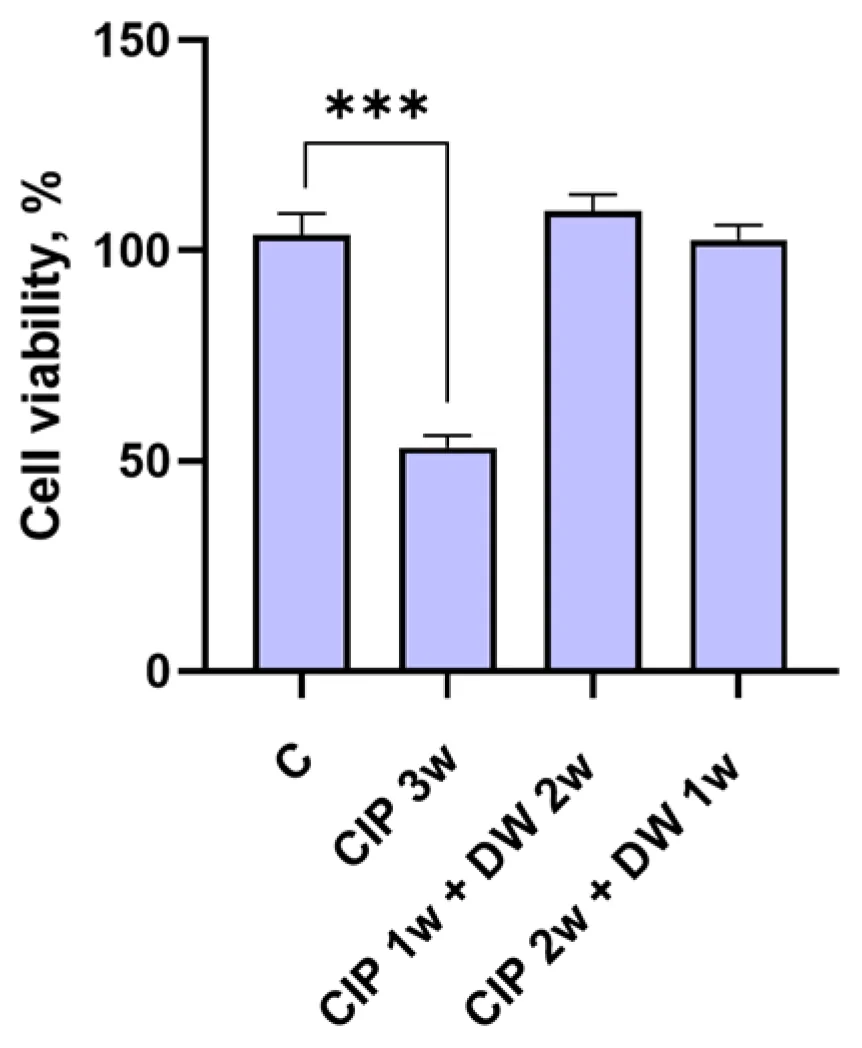
Recovery of cell viability after ciprofloxacin withdrawal in primary human fibroblasts. Primary human fibroblasts were exposed to 200 µM ciprofloxacin (CIP) continuously for 3 weeks (CIP 3w) or treated with CIP for 1 or 2 weeks followed by drug withdrawal (DW) and culture in CIP-free medium for an additional 2 or 1 weeks, respectively (CIP 1w + washout 2w and CIP 2w + washout 1w). Cell viability was assessed using the resazurin (Alamar Blue) assay by measuring resorufin fluorescence and expressed as a percentage of the untreated control (C). Data are presented as mean ± SEM; n = 3. Statistical analysis was performed by one-way ANOVA with Dunnett’s post hoc test (each treatment vs. control). *** *p* < 0.001 vs. control.

To determine whether the effect of prolonged CIP exposure persisted after drug withdrawal, we performed washout experiments. The fibroblasts for this experiment were initially seeded at ~50% confluency. Continuous CIP exposure for 3 weeks reduced resazurin fluorescence to approximately 50% of the untreated control (Figure 10). In contrast, cells exposed to CIP for 1 week followed by a 2-week drug-free period, or for 2 weeks followed by a 1-week drug-free period, exhibited resazurin signals comparable to those of untreated cells. Consistent with these measurements, routine bright-field microscopy revealed a visibly lower cell density after continuous 3-week CIP exposure, whereas cells cultured in CIP-free medium after drug withdrawal progressively repopulated the culture surface and reached an approximately confluent monolayer by the end of the recovery period. Together, these observations suggest that the growth arrest induced by prolonged CIP exposure is not maintained after CIP removal.

## 3. Discussion

The present study demonstrates that prolonged exposure to a high concentration of ciprofloxacin induces a non-canonical senescence-like phenotype in human fibroblasts. This phenotype was associated with oxidative stress and was attenuated by both a conventional antioxidant and a mitochondria-targeted antioxidant, supporting a mechanistic contribution of redox imbalance. CIP also increased SA-β-Gal activity in H9c2 rat cardiomyoblasts and human EA.hy926 endothelial cells, two cell lines previously used in senescence research [21,22].

Mitochondrial dysfunction and oxidative stress are established components of fluoroquinolone cytotoxicity [7,8,9,10,11,23], while mitochondrial dysfunction and increased ROS production are also closely linked to the induction and maintenance of cellular senescence [24]. The intersection of these processes during fluoroquinolone exposure, however, has received limited attention. Prolonged CIP exposure has previously been reported to induce a senescence phenotype in glioblastoma cells [17]. More recently, CIP was shown to promote placental senescence and pregnancy loss in a mouse model through a pathway involving TRIM21-mediated MFF degradation [18]. Our findings extend these observations to primary human fibroblasts and indicate that CIP-induced oxidative stress may participate in a sustained stress-response program with multiple features of senescence-like state. CIP also increased TRIM21 expression in our model (Figure 2e), raising the possibility that partially shared stress-response mechanisms operate across different cell types.

The absence of a prominent inflammatory SASP does not exclude a senescence-like state. CIP induced DNA damage response, stress-response genes, SA-β-Gal activity, increased cell size and autofluorescence, and reduced RB phosphorylation without eliciting a broad pro-inflammatory transcriptional or secretory program at 7 days (Figure 1, Figure 2, Figure 3, Figure 4 and Figure 9). SASP composition is highly heterogeneous and depends on cell type, inducing stimulus, and senescence subtype. Recent single-cell analyses likewise demonstrate substantial phenotypic heterogeneity among senescent human dermal fibroblasts [25].

The selective effect of CIP on senescence-like phenotypes suggests that pro-senescent activity is not a uniform property of fluoroquinolones (Figure 1). Although CIP, MOX, and LEV share a common antibacterial mechanism, they differ in chemical structure, cellular uptake, tissue distribution, mitochondrial effects, and their capacity to induce genotoxic or oxidative stress. These differences may account for the distinct cellular responses observed under otherwise comparable exposure conditions. The absence of a senescence-like response to LEV should be interpreted cautiously. Our data do not exclude a potential pro-senescent effect of levofloxacin under more strongly cytotoxic or otherwise exposure-matched conditions.

The reduction in RB phosphorylation at Ser795 observed after CIP treatment occurred without robust increases in p53, p16, or p21 protein levels (Figure 9). However, the present data do not establish the mechanism responsible for this change or its functional consequences. Unchanged total p53 does not exclude activation of the p53 pathway, which may occur through post-translational modifications and/or changes in subcellular localization. Total RB abundance, CDK activity, E2F signaling, and PP2A activity were not assessed in this study. Oxidative and genotoxic stress have previously been reported to promote PP2A-dependent dephosphorylation of RB family proteins [26], making this pathway one possible explanation for the observed decrease in RB phosphorylation. However, its involvement in the response to CIP remains speculative. Further studies are required to determine whether altered RB signaling contributes to CIP-induced growth suppression.

To our knowledge, this study provides the first evidence that antioxidant intervention can attenuate CIP-induced senescence-like changes together with the associated accumulation of a DNA damage marker (Figure 6 and Figure 8). This result is consistent with previous studies showing that antioxidants mitigate several forms of fluoroquinolone toxicity [7,9]. Dexamethasone has also been reported to suppress oxidative stress associated with fluoroquinolone-induced and age-related tendinopathy through upregulation of the antioxidant enzyme GPX3 [27]. In addition, the mitochondria-targeted antioxidant MitoQ protects human Achilles tendon cells against fluoroquinolone-induced oxidative stress and mitochondrial membrane damage [8].

Overall, both Trolox and SkQ3 attenuated several redox-related changes induced by CIP (Figure 7). SkQ3 nevertheless increased DCF fluorescence despite reducing DHE fluorescence and decreasing the MitoCLox signal (Figure 7b,c). Given the limited chemical specificity of DCFH-DA, this apparently discordant response may reflect alterations in intracellular redox chemistry, peroxidase-dependent probe oxidation, or other redox-active intermediates rather than a generalized increase in oxidative stress. Importantly, neither DCFH-DA nor bulk DHE fluorescence identifies a specific reactive species or its intracellular source. Thus, these measurements should be interpreted as indicators of altered cellular redox state rather than direct quantitative measurements of individual ROS. The absence of a detectable CIP-induced increase in the MitoCLox signal further argues against substantial mitochondrial lipid peroxidation under the experimental conditions used.

The washout experiments further indicate that at least some effects of prolonged CIP exposure are reversible after drug removal (Figure 10). The concordance between metabolic recovery measured by resazurin and the increase in cell density argues against the possibility that the restored resazurin signal simply reflects increased metabolic activity of a persistently growth-arrested cell population. Instead, the observations are consistent with at least partial recovery of proliferative capacity after CIP withdrawal. Nevertheless, cell density was assessed qualitatively, and the present experiments did not directly measure DNA synthesis or cell-cycle re-entry. 

Classical cellular senescence is generally associated with persistent cell-cycle arrest even after removal of the initiating stress. The recovery observed after CIP withdrawal, together with the absence of canonical SASP and the lack of marked p16, p21, or p53 protein induction, supports a more cautious interpretation. Apparently, CIP induces a reversible, non-canonical senescence-like state that may overlap phenotypically with stress-induced quiescence.

Whether the pro-senescent activity observed here contributes to fluoroquinolone adverse effects in vivo remains unknown. The capacity of therapy-induced senescent cells to promote systemic dysfunction, however, is well established in other settings. In vivo exposure to senescence-inducing agents such as doxorubicin can produce widespread toxicity with features resembling accelerated aging [28], including cardiac and vascular dysfunction, bone loss, renal fibrosis, liver injury, ovarian damage, fatigue, reduced physical activity, and impaired tissue regeneration [29]. Increased senescence markers have been detected in affected tissues, and genetic or pharmacological depletion of senescent cells can partially restore several functional outcomes, supporting a causal role for therapy-induced senescence [29]. These observations provide biological plausibility for the hypothesis that accumulation of senescence-like cells could contribute to some persistent effects of CIP, although this possibility requires direct in vivo testing.

The study has several limitations, including:

1. High fluoroquinolone concentrations used, which may not reproduce typical systemic exposure in vivo. The concentrations used here exceed the peak plasma concentrations generally achieved after standard intravenous dosing, and reported C_max_ values are approximately 4.3–4.6 mg/L (13–14 µM) after 400 mg intravenous ciprofloxacin, 3.9–5.1 mg/L (10–13 µM) after 400 mg intravenous moxifloxacin, and 6.2–11.5 mg/L (17–32 µM) after 500–750 mg intravenous levofloxacin [30,31,32]. Plasma C_max_, however, may not fully represent tissue-specific or intracellular exposure in compartments where fluoroquinolones accumulate or undergo active transport. CIP concentrations in some tissues can exceed those in serum [33], although the local concentrations relevant to prolonged cellular exposure remain uncertain [34]. Ciprofloxacin crystalluria and crystal nephropathy provide indirect evidence that high local drug concentrations can occur in the urinary tract and kidney, occasionally resulting in acute kidney injury [35]. These considerations do not eliminate the concentration limitation of the present model but indicate that plasma exposure alone may not capture all clinically relevant local conditions. Accordingly, the present experimental conditions should be considered a mechanistic model of prolonged exposure with relatively high concentrations of the fluoroquinolones rather than a direct reproduction of clinically achieved systemic fluoroquinolone concentrations.

2. Administration of fluoroquinolones as commercial intravenous infusion solutions, which contain excipients (sodium chloride, lactic acid, and disodium edetate in the ciprofloxacin formulation). Although the final excipient concentrations at the working dilutions were low (e.g., 0.24 mM lactic acid, 0.018 mM EDTA), the absence of a vehicle control group treated with the excipients alone precludes formal exclusion of minor excipient contributions. However, the selectivity of the senescence-like phenotype—induced by CIP but not by MOX or LEV, despite all three being prepared from infusion solutions containing sodium chloride—strongly argues that the observed effects are attributable to ciprofloxacin rather than to shared excipients.

3. Primary human fibroblasts used in this study were derived from only three healthy male donors. While the consistency of the CIP-specific effect across all three donors and across two additional cell lines (EA.hy926 and H9c2) provides evidence that the observed phenotype is not idiosyncratic to a single donor, this cohort size precludes robust assessment of inter-individual variability and does not capture potential sex-specific differences in senescence and oxidative stress responses. Future studies should include fibroblasts from female donors, a larger donor cohort, and donors spanning a range of ages.

4. It should also be noted that cells were cultured in high-glucose DMEM (25 mM D-glucose), a condition that has been reported to induce oxidative stress and cellular senescence in certain cell types. However, several considerations indicate that this is unlikely to have confounded our findings. In retinal endothelial cells, which appear more susceptible to glucose-induced senescence than fibroblasts, continuous culture in 25 mM glucose required 4 weeks to accelerate the senescence program, as evidenced by increased SA-β-Gal positivity and growth arrest [36]. In human dermal fibroblasts, induction of senescence by high glucose within a 7-day period required a concentration of 35 mM, substantially above the level used in standard high-glucose DMEM [37]. Consistent with these observations, our untreated control cells maintained in high-glucose DMEM for 7 days did not exhibit significantly elevated SA-β-Gal activity, or senescent morphology. Nevertheless, we cannot exclude the possibility that high glucose may have sensitized cells to ciprofloxacin-induced oxidative stress, potentially modulating the magnitude of the observed response.

5. A further limitation is that apoptotic markers were not directly assessed in this study. The distinction between cellular senescence and apoptosis requires evaluation of multiple markers, as the two processes can share overlapping features and may coexist within a treated cell population. CIP has been reported to induce apoptosis in certain cell types, including breast cancer and glioblastoma cells, via mitochondrial pathways involving caspase-3/7 activation and Bax/Bcl-2 signaling, and to cause cytotoxicity associated with lipid peroxidation and glutathione depletion in human fibroblasts [38,39]. Although several lines of indirect evidence argue against apoptosis being the primary phenotype in our model—including the persistence of SA-β-Gal-positive, enlarged cells over 7–14 days, the stability of the phenotype in adherent cells, and its attenuation by antioxidant co-treatment—the absence of direct apoptosis measurements precludes definitive exclusion of apoptotic contributions.

Further studies are required to define the stability, mechanism, and biological relevance of the CIP-induced senescence-like phenotype. Broader transcriptomic, proteomic, and metabolomic profiling could clarify how this non-canonical state differs from classical DNA damage-induced senescence, mitochondrial dysfunction-associated senescence, quiescence, and other forms of stress adaptation. Cell-type specificity also requires further investigation, as our data are derived primarily from human fibroblasts, with additional observations in endothelial cells and cardiomyoblasts. Because fluoroquinolone-associated adverse effects frequently involve connective tissue, skeletal muscle, tendons, peripheral nerves, and the cardiovascular system, relevant models should include tenocytes, neuronal or glial cells, and ultimately animal models. Such studies will help determine whether CIP-induced oxidative stress and senescence-like remodeling represent a mechanistically relevant contributor to persistent tissue dysfunction.

## 4. Materials and Methods

### 4.1. Materials and Reagents

MitoCLox, a ratiometric fluorescent probe sensitive to lipid peroxyl radicals, was synthesized from the succinimidyl ester of C11-BODIPY581/591 and (5-[(4-aminobutyl)amino]-5-oxopentyl)triphenylphosphonium bromide as described previously [40]. SkQ3 was kindly provided by LLC “Institute of Mitoengineering, MSU”. CM-H2DCFDA, and dihydroethidium (DHE) were purchased from Invitrogen Life Technologies (Waltham, MA, USA). LumiTracker^®^ Lyso Red was from Lumiprobe Corporation (Hannover, Germany). Unless otherwise indicated, all other reagents were obtained from Sigma-Aldrich (St. Louis, MO, USA).

### 4.2. Cell Cultures and Fluoroquinolone Treatment

Rat H9c2 cardiomyoblasts, a spontaneously immortalized cell line (EcACC Cat. No. 88092904), and the human endothelial cell line EA.hy926 were from ATCC collection (Cat. No. CRL-2922), and primary human skin fibroblasts were from three healthy male volunteers obtained from the Common Use Center “Biobank” (Research Centre for Medical Genetics, Moscow, Russia). The donors were healthy adults aged 28–35 years with no known chronic diseases. The fibroblasts were cultured in Dulbecco’s modified Eagle’s medium (DMEM, 4.5 g/L D-glucose, 25 mM; Gibco, Thermo Fisher Scientific, Waltham, MA, USA) supplemented with 2 mM glutamine and 10% fetal bovine serum (FBS; HyClone, Logan, UT, USA). Experiments were performed at passages comprised between 2 and 7. Cells were seeded in experimental plates at approximately 40% confluence and allowed to reach 50–60% confluence before treatment, unless indicated otherwise. Antibiotics and antioxidants were then added for the indicated exposure periods and were not replenished during incubation. For the positive-control condition, DOX was removed after 24 h by replacing the medium with fresh DOX-free medium. Cell confluence reached approximately 100% by the end of the treatment period. In the 14-day experiments, cells were not passaged, and the culture medium was not replaced after seeding. Ciprofloxacin, moxifloxacin, and levofloxacin were obtained as commercial solutions for intravenous infusion from PJSC KrasPharma (Krasnoyarsk, Russia). The ciprofloxacin infusion solution (2 mg/mL) contained sodium chloride (9 mg/mL), lactic acid (0.65 mg/mL), and disodium edetate dihydrate (0.20 mg/mL) as excipients (pH 3.5–4.6). The moxifloxacin infusion solution (1.6 mg/mL) contained sodium chloride (8 mg/mL) as the sole excipient (pH 4.1–4.6). The levofloxacin infusion solution (5 mg/mL) contained sodium chloride (9 mg/mL) as the sole excipient (pH 4.3–5.3). At the working concentrations used in this study (200 μM CIP, 200 μM MOX, 500 μM LEV), the final concentrations of excipients in the culture medium were: NaCl, 5.1–6.9 mM (added to ~110 mM already present in DMEM); lactic acid, 0.24 mM (CIP only); and disodium edetate, 0.018 mM (CIP only). The pH of the culture medium was not altered by the addition of the infusion solutions at these dilutions, as expected from the ~20–30-fold dilution in bicarbonate-buffered DMEM and confirmed by visual inspection of phenol red color. Cells with no treatment were used as control cells.

### 4.3. Detection of SA-β-Galactosidase (SA-β-Gal) Activity

SA-β-Gal activity was measured using a histochemical staining kit (Servicebio, Wuhan, China, Cat. No. G1073-100T) according to the manufacturer’s instructions. Cells were fixed and incubated with X-Gal staining solution at pH 6.0 at 37 °C for 4–12 h (the presence or absence of staining was regularly monitored using microscopy). Incubation time was the same for each condition within biological replicate. Bright-field images of SA-β-Gal-stained cells and corresponding Hoechst 33342 nuclear images were captured using an Olympus IX83 microscope (Olympus Corporation, Tokyo, Japan). For each condition, 3 (for 4× magnification) or 10 (for 20× magnification) randomly selected fields were imaged per biological replicate. ImageJ (NIH) was used to measure the integrated density of the SA-β-Gal signal in each field, and the nuclear count was determined from the Hoechst image of the same field. SA-β-Gal activity was expressed as integrated density per cell (integrated density/nuclear count). Multiple fields from each sample were averaged to obtain one value per biological replicate. The same quantification procedure and unit notation were applied to all cell types (primary human fibroblasts, EA.hy926, and H9c2) across all figures.

### 4.4. Measurement of Cell Viability/Cytotoxicity by Resazurin Assay

Cell viability/cytotoxicity was assessed by the resazurin reduction assay using CellTiter-Blue^®^ reagent (Promega, Madison, WI, USA) according to the manufacturer’s protocol. Fluorescence was measured with a Fluoroskan Ascent FL microplate reader (Thermo Labsystems, Waltham, MA, USA).

### 4.5. Flow Cytometry Measurements

Flow cytometric measurements were performed using an Amnis^®^ FlowSight^®^ Imaging Flow Cytometer (Luminex, Austin, TX, USA) provided by the Moscow State University Development Program PNR5. Cells were detached with trypsin/Versene solution (1:1), centrifuged at 1000× *g* for 5 min at 4 °C, and resuspended in 50 µL PBS. Mean fluorescence intensity (MFI) was measured on a per-cell basis for all probes. Data were analyzed using IDEAS software version 6.3 (Luminex). The gating cascade was applied in the following order: (1) single cells were selected using a plot of bright-field area versus aspect ratio to exclude doublets and debris; (2) focused cells were selected from the single-cell population using the gradient RMS feature of the bright-field image, with a threshold of 50 to exclude out-of-focus events (gradient RMS is a texture-based sharpness metric computed as the root mean square of the local intensity gradient across image pixels); and (3) the final gated population, defined as the intersection of the single-cell and focus gates, was used for all downstream fluorescence measurements. The same gating template and thresholds were applied identically to all experimental groups within each experiment. Gates were defined on the control (untreated) population and applied uniformly to all samples. The representative plots for the imaging flow cytometry analyses are provided in Supplementary Figure S4.

### 4.6. Measurement of Senescence Index (SI)

The senescence index was determined by flow cytometry from the increases in cell size and lipofuscin-associated autofluorescence characteristic of senescent cells, as described previously [19]. SI is an empirical composite index calculated from normalized cell diameter (nD) and normalized autofluorescence (nAF), relative to proliferating control cells, using the formula: SI = ((nAF − 1) + 5 × (nD − 1))/2. The weighting factor of 5 for the diameter reflects the empirical observation that senescent cells exhibit much smaller increase in diameter (~10%) rather than increase in autofluorescence (~50%), so the 5× multiplier is introduced to equalize the contribution of the two parameters. Dividing to a coefficient of 2 is necessary to balance the contributions of the two parameters.

### 4.7. Measurement of Lysosomal Mass

To label lysosomes, primary human fibroblasts were incubated with 50 μM LumiTracker^®^ Lyso Red for 15 min before flow cytometric analysis.

### 4.8. Measurement of Oxidative Stress

For oxidative stress measurements, primary human fibroblasts were incubated with 2 μM DCFH-DA or 2 μM DHE for 30 min before flow cytometric analysis. The mitochondria-targeted ratiometric fluorescent probe MitoCLox, which is sensitive to lipid peroxyl radicals, was used as described previously [40]. For all probes, unstained cells (without probe) were analyzed in parallel to establish baseline autofluorescence, and this background was subtracted from probe-treated samples. Cells treated with the probes but without fluoroquinolone served as the untreated control. For MitoCLox, which is a ratiometric probe (520/590 nm fluorescence ratio), the ratio inherently corrects for variable probe loading, as both the oxidized and reduced forms of the probe are measured simultaneously in the same cell.

### 4.9. Western Immunoblotting

After incubation with the indicated compounds, cells were lysed in hot sample buffer (62.5 mM Tris-HCl, pH 6.8; 2% SDS; 10% glycerol; 50 mM DTT; 0.01% bromophenol blue) for 5 min at 94 °C. Proteins were separated by SDS-PAGE and transferred to a PVDF membrane (Bio-Rad, Hercules, CA, USA). Nonspecific binding sites were blocked with 5% BSA. Membranes were incubated overnight at 4 °C with primary antibodies diluted in 5% BSA in TBST (25 mM Tris, pH 7.4; 0.15 M NaCl; 0.1% Tween 20), followed by HRP-conjugated secondary antibodies in 5% BSA/TBST for 1 h at room temperature. Chemiluminescence was developed using West Dura Extended Duration Substrate (Thermo Fisher Scientific, USA), and images were acquired with a ChemiDoc™ gel imaging system (Bio-Rad, USA). Densitometric analysis was performed using Image Lab software v.6.0.1. Signals for proteins of interest were normalized to total protein content measured by 2,2,2-trichloroethanol fluorescence as described in [41,42].

### 4.10. RNA Isolation and Quantitative Real-Time PCR (RT-qPCR)

Total cellular RNA was isolated using the Quick-RNA MiniPrep kit (Zymo Research, Irvine, CA, USA) according to the manufacturer’s protocol. RNA concentration was determined with a NanoDrop ND-1000 spectrophotometer (Thermo Fisher Scientific, USA), and RNA quality was verified by electrophoresis. Reverse transcription was performed using the REVERTA-L reagent kit (AmpliSens, Moscow, Russia) according to the manufacturer’s protocol. The resulting cDNA was analyzed by real-time PCR using EvaGreen I intercalating dye (Syntol, Moscow, Russia). The following primers were used: *IL-6* (forward: 5′-GACAACTCATCTCATTCTG-3′; reverse: 5′-CCATTAACAACAACAATCTG-3′); *CXCL2* (forward: 5′-AACTGCGCTGCCAGTGCT-3′; reverse: 5′-CCCATTCTTGAGTGTGGCTA-3′); *GDF15* (forward: 5′-GTTCCTGAGACACCCGATT-3′; reverse: 5′-AATACACAGTTCCATCAGACCA-3′); *ANKRD1* (forward: 5′-AGTAGAGGAACTGGTCACTGG-3′; reverse: 5′-TGGGCTAGAAGTGTCTTCAGAT-3′); *TRIM21* (forward: 5′-ACCCCCTAAAGGTCTCCACA-3′; reverse: 5′-ATCATTGTCAAGCGTGCTGC-3′); *p16* (CDKN2A) (forward: 5′-GGGGGCACCAGAGGCAGT-3′; reverse: 5′-GGTTGTGGCGGGGGCAGTT-3′); *p21* (CDKN1A) (forward: 5′-GACACCACTGGAGGGTGACT-3′; reverse: 5′-CAGGTCCACATGGTCTTCCT-3′); *p53* (*TP53*) (forward: 5′-ACCACCATCCACTACAACTACAT-3′; reverse: 5′-ACAAACACGCACCTCAAAGC-3′); *GAPDH* (forward: 5′-GATGACATCAAGAAGGTGGTG-3′; reverse: 5′-GCTGTAGCCAAATTCGTTGTC-3′); *ACTB* (forward: 5′-CATCGAGCACGGCATCGTCA-3′; reverse: 5′-TAGCACAGCCTGGATAGCAAC-3′); *RPL32* (forward: 5′-CATCTCCTTCTCGGCATCA-3′; and reverse: 5′-AACCCTGTTGTCAATGCCTC-3′). Each sample was analyzed in triplicate (technical replicates) and the values were averaged before normalization, and a no-template control was included in each run. PCR efficiency (E) was determined from standard curves using the equation E = 10^(−1/slope)^. Target gene mRNA levels were normalized to three reference genes: *RPL32*, *ACTB*, and *GAPDH* according to [43].

### 4.11. Immunocytochemistry

Cells were seeded on glass coverslips in 6-well plates at 100,000 cells per well. After washing with DMEM, cells were fixed with 2% paraformaldehyde in DMEM for 10 min at 37 °C and permeabilized with 0.2% Triton X-100 in PBS for 5 min at room temperature. Cells were incubated overnight at 4 °C with a monoclonal anti-phospho-Histone H2AX (Ser139) antibody (sc-517348; Santa Cruz Biotechnology, Dallas, TX, USA). Secondary staining was performed for 1 h at room temperature using cross-adsorbed goat anti-mouse IgG (H+L) conjugated to Fluor647 (S0014; Affinity Biosciences, Changzhou, China) together with Hoechst 33342 (Biotium, Fremont, CA, USA). After washing with PBS, coverslips were mounted using Aqua-Poly/Mount (Polysciences, Warrington, PA, USA). Fluorescence microscopy was performed using an Olympus IX83 microscope (Olympus Corporation, Tokyo, Japan). Quantification of γH2AX foci was performed using Fiji/ImageJ2 software Version: 2.16.0/1.54p. Cell nuclei were segmented based on Hoechst 33342 fluorescence. Background fluorescence was subtracted from the γH2AX channel, followed by application of an identical intensity threshold to all experimental groups. γH2AX-positive cells were identified by visual scoring of nuclei with γH2AX staining clearly above background. Individual γH2AX-positive foci located within the boundaries of each nucleus were identified and counted using particle analysis. The number of γH2AX foci was determined separately for each nucleus, and the mean number of foci per nucleus was calculated for each experimental group. For each experimental group, γH2AX foci were quantified in 10 randomly selected fields of view in each of the three independent experiments. Images from all groups were acquired and analyzed using identical acquisition and image-processing parameters.

### 4.12. Enzyme-Linked Immunosorbent Assay (ELISA)

Relative levels of IL-1β, IL-6, and IL-8/CXCL8 in culture supernatants from primary human fibroblasts were quantified using commercial ELISA kits (Vector-Best, Koltsovo, Russia) according to the manufacturer’s instructions. Before analysis, supernatants were centrifuged at 3000× *g* for 10 min.

### 4.13. Statistical Analysis

Unless otherwise indicated, n refers to the number of independent biological replicates (i.e., independent experiments performed with cells from different donors or independent cell preparations). For each experiment, cells from each donor were seeded independently and treated in parallel. No experiment was repeated using cells from the same donor at different passages to generate additional “biological replicates” unless otherwise indicated. Technical replicates (e.g., triplicate qPCR reactions, multiple microscopy fields per sample) were averaged to yield a single value per biological replicate before statistical analysis. For qPCR, ELISA, and flow cytometry the number of technical replicates is 3. For Western immunoblotting the number of technical replicates is 2. In microscopy, unless the opposite mentioned, the number of technical replicates (different fields of view within a single biological replicate) varies depending on the magnification at which the image was captured: for 4× magnification, the number of fields of view was 3; for 20× magnification, it was at least 10. The number of biological replicates (n) and the statistical tests used for each experiment are specified in the corresponding figure legends. Also, unless otherwise indicated, data are presented as mean ± SEM. Statistical analyses and graph preparation were performed using GraphPad Prism 9 (GraphPad Software, San Diego, CA, USA). Given the small sample size (n = 3 per group), formal normality and variance homogeneity testing have insufficient statistical power to be informative. Parametric one-way ANOVA was used based on the conventional assumption of approximately normal distributions for in vitro quantitative data and on the robustness of ANOVA to moderate deviations from normality and variance heterogeneity in equal group sizes. One-way ANOVA was followed by Dunnett’s post hoc test for comparisons of each treatment group against the untreated control, and Bonferroni-corrected pairwise comparisons for comparisons between CIP-treated and antioxidant co-treated groups. A *p* value < 0.05 was considered statistically significant. Because each biological replicate corresponds to a different donor, the reported variability (SEM) reflects inter-donor variability. Formal modeling of donor-to-donor variability (e.g., mixed-effects analysis) was not performed due to the limited sample size.

## 5. Conclusions

Prolonged ciprofloxacin exposure induces an oxidative stress-associated, non-canonical senescence-like phenotype in human fibroblasts in vitro, accompanied by growth suppression that is reversible after drug withdrawal. The attenuation of γH2AX accumulation and multiple senescence-associated readouts by Trolox and SkQ3 supports a contribution of redox imbalance to this cellular response.

## Data Availability

The original contributions presented in this study are included in the article/Supplementary Materials. Further inquiries can be directed to the corresponding author.

## Supplementary Materials

The following supporting information can be downloaded at: https://www.mdpi.com/article/10.3390/ijms27198696/s1.

## Abbreviations

The following abbreviations are used in this manuscript:

BF	Bright-field
BSA	Bovine serum albumin
CDK	Cyclin-dependent kinase
CIP	Ciprofloxacin
C_max_	Maximum plasma concentration
DCFH-DA	2′,7′-Dichlorodihydrofluorescein diacetate
DHE	Dihydroethidium
DMEM	Dulbecco’s modified Eagle’s medium
DOX	Doxorubicin
γH2AX	Phosphorylated histone H2AX
LEV	Levofloxacin
MFI	Mean fluorescence intensity
MOX	Moxifloxacin
PP2A	Protein phosphatase 2A
RB	Retinoblastoma protein
ROS	Reactive oxygen species
RT-qPCR	Reverse transcription quantitative real-time PCR
SA-β-Gal	Senescence-associated β-galactosidase
SASP	Senescence-associated secretory phenotype
SI	Senescence index
