# Supplementary material for: Ciprofloxacin-Induced Oxidative Stress Promotes a Senescence-like Phenotype in Human Fibroblasts In Vitro
Source: Int J Mol Sci. 2026 Sep 29;27(19):8696. doi: 10.3390/ijms27198696 (PMC13664843; doi:10.3390/ijms27198696)
Supplement: Supplementary file 1 [file ijms-27-08696-s001.zip › Supplementary Figure S1.pdf]

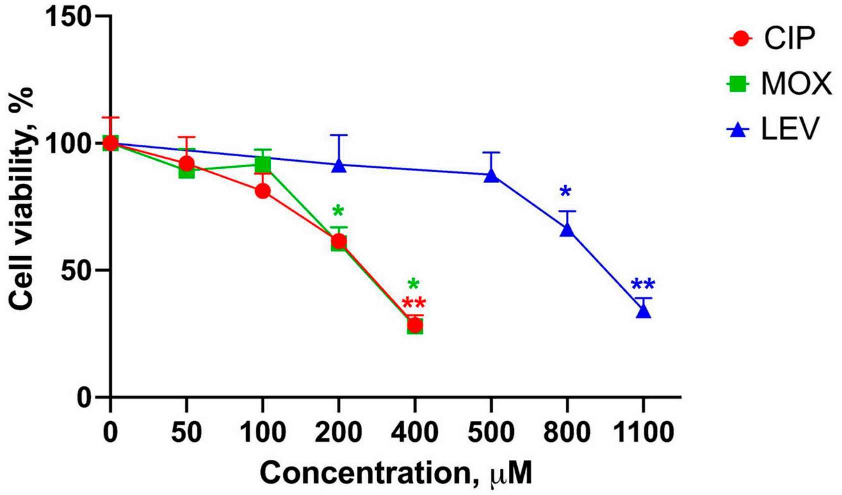

Supplementary Figure S1. Effects of ciprofloxacin (CIP), moxifloxacin (MOX), and levofloxacin (LEV) on the viability of primary human fibroblasts. Cells were treated for 7 days with CIP at 50, 100, 200, or 400 μM; MOX at 50, 100, 200, or 400 μM; or LEV at 200, 500, 800, or 1100 μM. Cell viability was assessed using the resazurin (Alamar Blue) assay by measuring resorufin fluorescence. Data are presented as mean ± SEM from n = 3 independent biological replicates per group and expressed as a percentage of the untreated control. Statistical analysis was performed by one-way ANOVA with Dunnett's post-hoc test (each treatment vs. control). *P < 0.05, ** P < 0.01 vs. control.
