# Supplementary material for: Ciprofloxacin-Induced Oxidative Stress Promotes a Senescence-like Phenotype in Human Fibroblasts In Vitro
Source: Int J Mol Sci. 2026 Sep 29;27(19):8696. doi: 10.3390/ijms27198696 (PMC13664843; doi:10.3390/ijms27198696)
Supplement: Supplementary file 1 [file ijms-27-08696-s001.zip › Supplementary Figure S2.pdf]

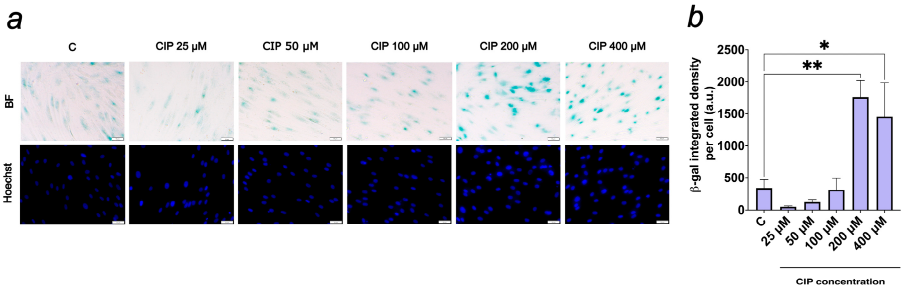

Supplementary Figure S2. Dose-dependent induction of SA-β-Gal activity in human fibroblasts treated with ciprofloxacin (CIP) for 7 days. (a) Representative bright-field images of senescence-associated β-galactosidase (SA-β-Gal) staining (upper row) and corresponding Hoechst 33342 nuclear staining (lower row) in primary human fibroblasts treated for 7 days with the indicated concentrations of CIP: 0, 25, 50, 100, 200, and 400 μM. Scale bar: 50 μm. (b) Quantification of SA-β-Gal activity expressed as integrated density per cell. Bright-field images were analyzed using ImageJ; integrated density of the SA-β-Gal signal in each field was divided by the nuclear count determined from the corresponding Hoechst image. Data are presented as mean of 3 independent experiments from 1 donor ± SEM. Statistical comparisons were performed using one-way ANOVA followed by Dunnett's post hoc test (each concentration vs. untreated control). $*P < 0.05$, $**P < 0.01$.
