# Supplementary material for: Ciprofloxacin-Induced Oxidative Stress Promotes a Senescence-like Phenotype in Human Fibroblasts In Vitro
Source: Int J Mol Sci. 2026 Sep 29;27(19):8696. doi: 10.3390/ijms27198696 (PMC13664843; doi:10.3390/ijms27198696)
Supplement: Supplementary file 1 [file ijms-27-08696-s001.zip › Supplementary Figure S3.pdf]

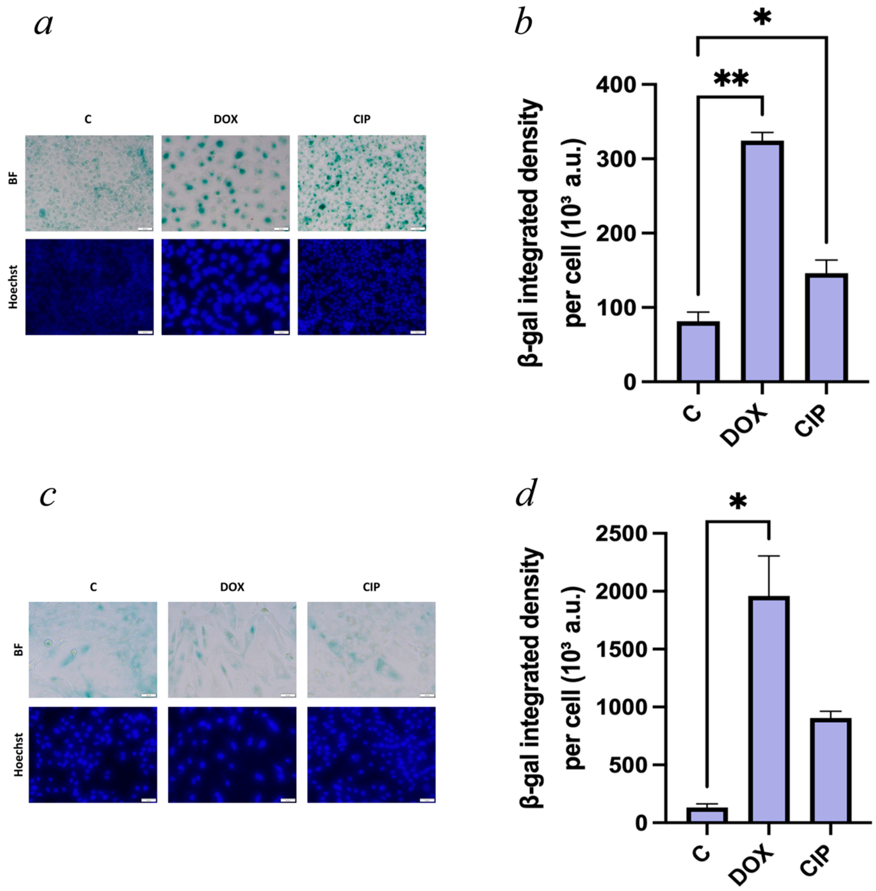

Supplementary Figure S3. Effects of ciprofloxacin (CIP) on senescence-associated β-galactosidase activity in EA.hy926 endothelial cells (**a, b**) and H9c2 cardiomyoblasts (**c, d**). (**a**) Representative images of SA-β-Gal and Hoechst staining in EA.hy926 cells treated for 7 days with 100 μM CIP or 50 μM MOX. Doxorubicin (DOX; 250 nM) was included as a positive control for senescence induction. Scale bar: 200 μm. (**b**) Quantification of SA-β-Gal staining as integrated density per cell, normalized to the nuclear count. Images were analyzed using ImageJ; multiple fields from each sample were averaged to obtain one value per biological replicate. Data are presented as mean ± SEM from n = 3 independent biological replicates per group. Statistical comparisons were performed using one-way ANOVA followed by Dunnett's post hoc test (each treatment vs. control). $^{*}P < 0.05$, $^{**}P < 0.01$ vs. control.
