# Supplementary material for: Ciprofloxacin-Induced Oxidative Stress Promotes a Senescence-like Phenotype in Human Fibroblasts In Vitro
Source: Int J Mol Sci. 2026 Sep 29;27(19):8696. doi: 10.3390/ijms27198696 (PMC13664843; doi:10.3390/ijms27198696)
Supplement: Supplementary file 1 [file ijms-27-08696-s001.zip › Supplementary Figure S4.pdf]

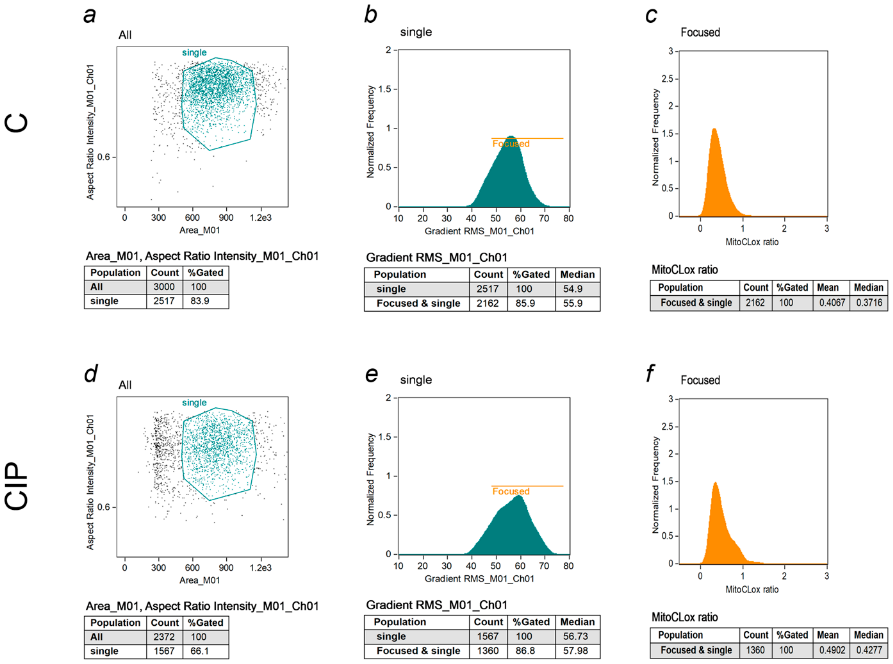

Supplementary Figure S4. Gating strategy for imaging flow cytometry analysis of primary human fibroblasts. Representative gating plots from an independent experiment are shown for untreated control cells (**a–c**) and cells treated with 200 μM ciprofloxacin (CIP) (**d–f**). (**a, d**) Single-cell gate: singlets were identified using cell area versus aspect ratio of the bright-field image to exclude doublets and debris. (**b, e**) Focus gate: cells in focus were selected from the single-cell population using the gradient RMS feature. (**c, f**) Final gated population used for all subsequent fluorescence intensity measurements. Identical gating thresholds were applied across all experimental groups. Data were acquired on an Amnis FlowSight imaging flow cytometer and analyzed using IDEAS software.
